# Effect of Whey Protein Isolate and Soy Protein Isolate on Textural Properties and Syneresis of Frozen Traditional Chinese Hot Pot Egg Sausage Gels

**DOI:** 10.3390/gels10120815

**Published:** 2024-12-11

**Authors:** Hong-Ting Victor Lin, Luan-Hui Huang, Jenn-Shou Tsai, Wen-Chieh Sung

**Affiliations:** 1 Department of Food Science, National Taiwan Ocean University, Keelung 202301, Taiwan; hl358@mail.ntou.edu.tw (H.-T.V.L.); allin981@yahoo.com.tw (L.-H.H.); tsaijs@mail.ntou.edu.tw (J.-S.T.); 2 Center of Excellence for the Oceans, National Taiwan Ocean University, Keelung 20231, Taiwan

**Keywords:** whey protein isolate, soy protein isolate, egg sausage gel, syneresis, modified cassava starch, sensory evaluation

## Abstract

Egg sausages, an essential component of traditional Chinese hot pot cuisine, have specific storage requirements and are predominantly distributed through refrigerated channels. A significant consideration in the freezing of egg sausages pertains to syneresis and textural modifications that manifest in the protein gel structure upon thawing. This research investigated the efficacy of incorporating whey protein isolate, soy protein isolate (at concentrations of 0.5%, 1.0%, and 2.0%), and modified cassava starch (at concentrations of 1.0%, 2.0%, and 3.0%) to enhance the textural integrity and mitigate syneresis in frozen egg sausage gels. The research demonstrated that syneresis in frozen egg sausages could be significantly minimized from 9.01% to 1.16% through the incorporation of 3% modified cassava starch and 2% whey protein isolate, to 2.01% with 1.0% soy protein isolate, and to 3.05% with 1.0% whey protein isolate. Furthermore, the combination of modified cassava starch (3%) and whey protein isolate (2%) demonstrated enhanced textural characteristics in frozen egg sausages with 20% additional water content following a 15-day storage period. Notably, egg sausages formulated with 0.5% whey protein isolate exhibited superior sensory attributes, including springiness, texture, and overall acceptability, compared to other formulations. The incorporation of whey protein isolate yielded markedly improved sensory characteristics relative to soy protein isolate additions. The findings indicate that the incorporation of whey protein isolate (0.5–1.0%) in conjunction with modified cassava starch (3%) effectively improves textural properties while reducing syneresis in thawed egg sausages.

## 1. Introduction

Egg sausage represents a traditional Chinese hot pot ingredient consisting of whole liquid egg, water, sugar, and salt encased in a pig’s small intestine, formed through heat-induced protein gel aggregation. These sausages are customarily prepared in uniform slices (2 cm height, 2.5 cm diameter) and require refrigeration to preserve quality and inhibit microbial proliferation. During storage, syneresis may occur—a phenomenon where water previously contained within the egg sausage gel matrix is released through contraction. This effect becomes notably pronounced following frozen storage, with commercial products typically reaching equilibrium after approximately ten hours of refrigeration. The product’s quality is primarily determined by its springiness, firmness, and chewiness, which are evaluated through standardized compression testing protocols.

The formation of syneresis and spongy texture in frozen egg sausages constitutes a significant quality concern, as water readily separates from the protein gel matrix upon thawing, resulting in an undesirable spongy consistency [1]. Consequently, these products are predominantly distributed through refrigerated channels and exhibit limited shelf stability.

The egg components serve as crucial constituents in the protein gel system during production. The three-dimensional gel structure is maintained through hydrogen bonding, disulfide cross-linking, and hydrophobic interactions during thermal processing. Egg yolk functions as an emulsifier, stabilizing oil droplets and foam formation in the liquid egg mixture [2]. The egg white proteins contribute additional functional properties, including gelation, emulsification, heat setting, and binding capabilities [3]. The gel formation process involves thermal-induced coagulation and denaturation of both yolk and albumen proteins [4]. This protein denaturation encompasses the disruption of hydrogen bonds, polypeptide chain unfolding, and exposure of reactive sites. Subsequently, these denatured proteins establish chemical and physical bonds, forming aggregates that contribute to the three-dimensional gel structure. The aggregation of denatured albumen proteins specifically involves hydrophobic, electrostatic, and disulfide bonds [5,6].

Protein–protein interactions are fundamental during both the gelation and storage phases [7,8]. The interaction between globular proteins can produce synergistic effects, including phase separation or precipitation, which influence textural characteristics based on their physicochemical properties and structural configurations [9,10]. This synergistic principle enables the development of enhanced protein gels using reduced protein concentrations. Soy and whey protein isolates have garnered significant attention due to their superior nutritional profiles, health benefits, and functional attributes [11]. These globular proteins, along with egg protein, are extensively utilized in dairy, bakery, and meat applications for water binding, cost reduction, nutritional enhancement, and texture improvement. Contemporary processed foods frequently incorporate hydrocolloids and starches to establish structural integrity and enhance sensory attributes [12,13]. Starch supplementation has demonstrated effectiveness in reducing syneresis and improving textural properties in surimi products [14]. Research indicates that hydrocolloid interactions within gel systems significantly enhance traditional food products, facilitate innovative food development, and optimize functional properties [15]. Polysaccharides contribute multiple functional benefits, including viscosity control, mouthfeel enhancement, protein stabilization during thermal processing, improved aeration properties, fat globule surface modification, acid stability, and freeze–thaw stability. Numerous food products incorporate polysaccharides, such as starch and carrageenan, to achieve desired sensory and textural characteristics [16].

Given the diverse nature of soy, whey, and egg proteins, a comprehensive evaluation of their compatibility and interactions is essential. The research hypothesis proposes that protein isolates and modified cassava starch will enhance texture stability and minimize syneresis in frozen egg sausage gels. Investigation of the physicochemical properties of these protein–starch systems has advanced our understanding of structure–function relationships, benefiting frozen food applications. Despite the potential applications, comprehensive studies focusing on egg sausage formulation remain limited. This research aims to evaluate the effects of whey protein isolate (0.5–2%), soy protein isolate (0.5–2%), and modified cassava starch (1–3%) incorporation on syneresis prevention and texture preservation in frozen egg sausage gels.

## 2. Results and Discussion

### 2.1. Textural Properties and Syneresis of Egg Sausages at Various Protein Isolate and Modified Cassava Starch Concentrations

This study examined the textural properties of egg sausage using various concentrations of whey protein isolate (0.5% and 1.0%) and modified cassava starch (1.0%, 2.0%, and 3.0%), as evaluated through penetration testing and presented in Table 1 and Table 2. Our analysis revealed that the incorporation of modified cassava starch and whey protein isolate led to significant reductions (*p* < 0.05) in all measured parameters of frozen egg sausages when tested at −18 °C for 15 days. This effect can be attributed to the formation of less robust heat-induced structures, resulting from the swelling of whey protein isolates and gelatinization of modified cassava starch within the egg protein gel matrix. Research by Pu et al. [17] established that egg albumen produces a more structured and robust gel network compared to whey isolate. When incorporated into the egg protein gel matrix, denatured whey protein isolate appears to influence the structural integrity, leading to reduced break point, breaking force, rigidity, and gel strength measurements [18]. Similarly, the gelatinized modified cassava starch integrates into the egg sausage gel network. These findings suggest that both gel rigidity and network water holding capacity are crucial determinants in assessing egg sausage stability against syneresis. According to McSwiney et al. [19], network formation occurs through the aggregation of unfolded whey protein molecules via nonspecific hydrophobic and sulfhydryl disulfide interactions. Our observations indicate that elevated concentrations of both whey protein isolate and modified cassava starch resulted in enhanced gel strength (Table 1 and Table 2).

Analysis of Table 1 and Table 2 reveals that frozen egg sausage containing modified cassava starch or whey protein isolate exhibits reduced breaking point, breaking force, and gel strength compared to samples stored at 4 °C for 24 h. The force–deformation curve analysis indicates elevated gel rigidity (initial slope of the curve) in frozen samples compared to refrigerated counterparts. This phenomenon can be attributed to protein denaturation and subsequent water loss from the protein gel matrix during frozen storage. The structural integrity is maintained through cross-linking between egg protein, sucrose, salt, and egg yolk fat components post-simmering. The product’s structure consists of a three-dimensional network capable of significant water retention, with egg proteins serving as the primary structural component. Post-freezing, the texture demonstrates reduced resistance to mastication. Our findings indicate that while modified cassava starch and whey protein isolate contribute to textural refinement in frozen samples, they do not exhibit significant synergistic interactions at the tested concentrations. Given the potential for adverse organoleptic properties at higher concentrations, whey protein isolate incorporation should be maintained at or below 2%.

**Figure 1:**
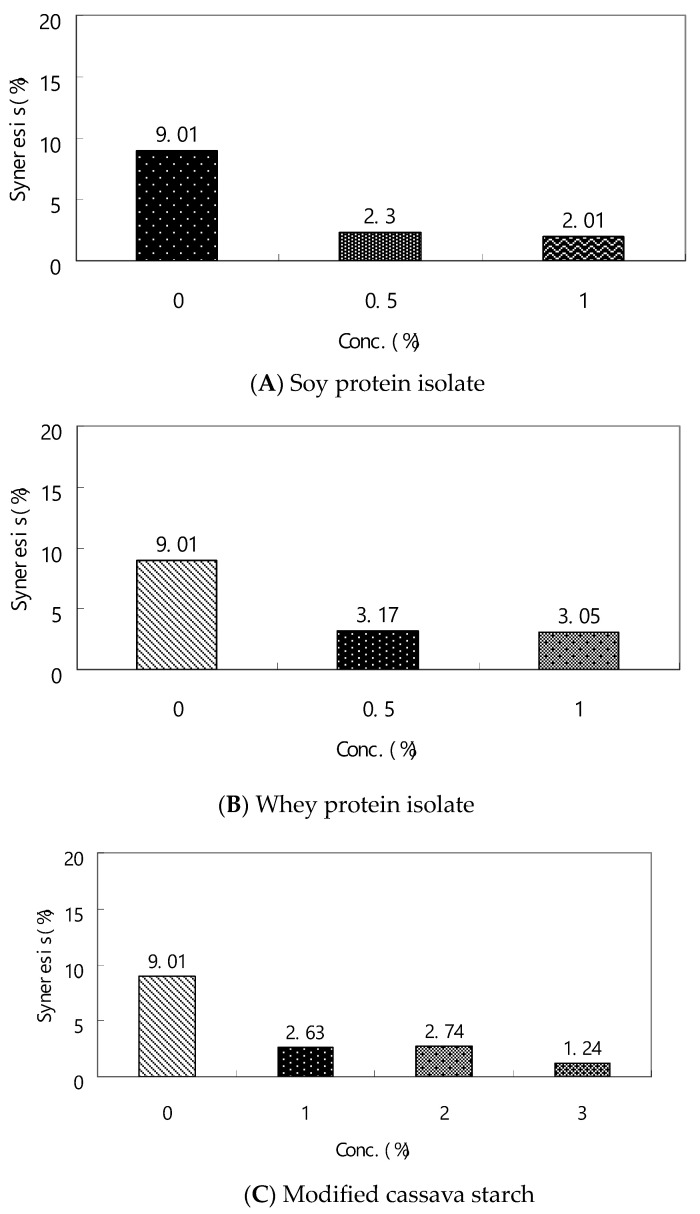
Effect of whey protein isolate, soy protein isolate, and modified cassava starch on syneresis of frozen egg sausage.

The percentage of syneresis in frozen egg sausage serves as a key metric for assessing how protein gels resist adverse physical alterations during freezing storage (Figure 1A,B). The phenomenon of syneresis in frozen egg sausage occurs due to enhanced molecular aggregation between protein molecules and internal bonds, particularly involving denatured hydrophobic side chains, resulting in water expulsion from the protein gel matrix. Consequently, the volume of released water serves as an effective indicator of protein denaturation during frozen storage. The separation of water from frozen egg sausage not only diminishes consumer acceptance and texture quality but may also result in the loss of water-soluble nutrients. Nevertheless, the incorporation of protein isolates and cassava starch, which demonstrate water retention capabilities, effectively reduces syneresis. Research has demonstrated that whey proteins possess the capacity to form heat-induced gels that effectively retain substantial quantities of water and other food components [20,21]. Syneresis manifests as a reorganization of the gel matrix through the enhancement of egg protein–whey protein isolate or soy protein isolate junctions, which is influenced by ingredient composition. The denatured whey proteins form hydrogen bonds with water molecules. As a result, the egg protein and whey protein isolate gel matrix exhibits reduced syneresis compared to egg sausage gel independently. The addition of 3% modified cassava starch demonstrated significant efficacy in reducing syneresis to 1.24% in frozen egg sausage (Figure 1C).

Table 3 presents the textural properties analysis of egg sausage containing soy protein isolate at 0.5% and 1.0% concentrations, as determined through penetration testing. The incorporation of soy protein isolate demonstrated no significant alterations in penetration test parameters of egg sausages stored at 4 °C for 24 h. Research by Comfort and Howell [22] demonstrated that elevated protein concentrations led to enhanced storage modulus in the soya isolate gel network. Their findings indicated an increase in intermolecular cross-links, resulting in a more cohesive matrix [22]. However, they noted that beyond certain protein concentration thresholds, the increase in storage modulus became less pronounced due to reduced protein solubility and matrix formation participation [22]. This observation potentially explains why the addition of whey protein isolate and soy protein isolate did not enhance the breaking force and gel strength of egg sausage in our current study (Table 2 and Table 3). This may be attributed to the established formation of the egg protein gel, which limits the solubility of whey protein isolate and soy protein isolate. While egg sausage is characterized as a three-dimensional protein network capable of water retention, the microstructural composition—comprising a protein network with embedded yolk fat globules or water-emulsified components—plays a crucial role. Previous research by Howell [9] has documented synergistic interactions and phase aggregation in protein mixtures, including egg albumen, whey, and soya. However, our findings indicate that soy protein isolate did not produce an enhanced synergistic effect in the egg protein–soy protein isolate combined systems. Analysis reveals that frozen egg sausage exhibits marginally higher rigidity compared to samples stored at 4 °C for 24 h (Table 3). This can be attributed to water displacement from the denatured protein gel matrix following frozen storage. Furthermore, while the breaking force of frozen egg sausage decreased, the breaking point showed minimal variation (*p* > 0.05) post-thawing (Table 3). Penetration test data indicate lower breaking force values in frozen egg sausage compared to refrigerated samples at 4 °C for 24 h, suggesting reduced textural firmness after frozen storage. The experimental results demonstrate that soy protein isolate reduces the breaking force and gel strength of egg sausage following frozen storage.

Analysis of Figure 1A indicates that soy protein isolate demonstrates superior water retention properties post-thawing compared to whey protein isolate (Figure 1B), effectively mitigating the syneresis phenomenon. The incorporation of these protein isolates enhances hydrophilic characteristics through increased protein content. During the freezing process, the volumetric expansion of water impacts the structural integrity of the egg protein–soy protein isolate gel matrix. The extent of syneresis in thawed egg sausages is determined by various gel permeability factors, including pore dimensions, protein–protein interactions, and the compositional heterogeneity within the egg sausage gel network. The structural integrity of the continuous egg protein network undergoes modification due to ice crystal formation and protein reorganization during freezing, resulting in increased porosity of the egg gel matrix, which facilitates water release upon thawing. Research has established that gellan gum exhibits gel-forming capabilities in both substituted and unsubstituted forms at minimal concentrations [23,24]. Research by Gibson and Sanderson [25] demonstrated that substituted form–soy protein isolate gel exhibits favorable textural properties upon thawing, producing gels with desirable softness and tenderness. Their findings indicate that the egg protein–soy protein isolate gel demonstrates excellent freeze tolerance, with soy protein isolate effectively mitigating ice crystal damage to the egg protein gel structure. While previous studies have shown that elevated concentrations of salt and sucrose (20%) can lower the freezing point and provide protective effects against frozen egg damage [24], our analysis revealed that moderate levels of salt (2%) and sucrose (1.5%) proved insufficient in protecting against freezing stress in egg sausage stored at −18 °C.

Table 4 presents the analysis of texture properties in egg sausages containing various whey protein isolate concentrations and 3% modified cassava starch. The incorporation of 3% modified cassava starch led to significant (*p* < 0.05) reductions in the breaking point of egg sausages from 9.60 mm to 8.16 mm when stored at 4 °C for 24 h (Table 2 and Table 4). Furthermore, this addition resulted in significant (*p* < 0.05) enhancements in both breaking force and rigidity under the same storage conditions (Table 2 and Table 4). As a thickening agent, starch represents an efficient carbohydrate composed of glucose units connected through extensive glycosidic bonds and branched molecular structures. During thermal processing, starch granules undergo swelling and water absorption, leading to gelatinization that enhances gel system viscosity and reduces syneresis. Research indicates that cassava starch gels demonstrate notably lower firmness compared to whey protein isolate gels at equivalent concentrations [26,27]. In bologna sausage applications, starch incorporation facilitates the development of a more consolidated and robust heat-induced protein matrix [28]. The combination of 3% modified cassava starch and 2% whey protein isolate demonstrates a beneficial synergistic interaction within protein–starch composite systems.

**Figure 2:**
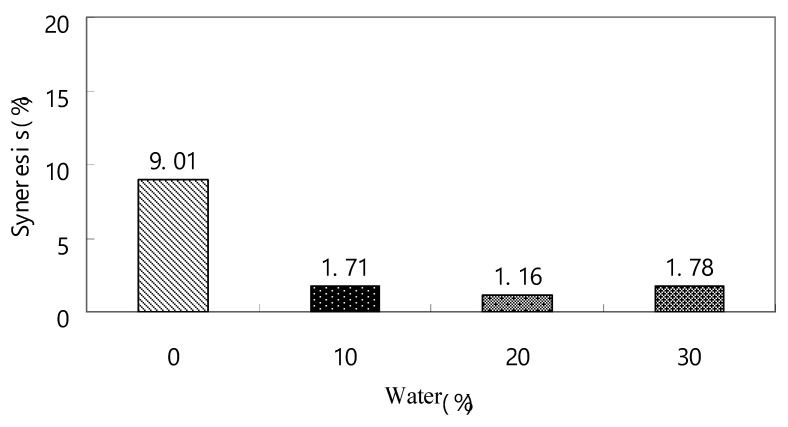
Effect of extra water addition (10% to 30%) on syneresis of frozen egg sausage containing 3% modified cassava starch and 2% whey protein isolate. Moreover, 0%, egg sausage without modified cassava starch and whey protein isolate added; 10%, egg sausage with 3% modified cassava starch and 1% whey protein isolate and extra 10% water added; 20%, egg sausage with 3% modified cassava starch and 1% whey protein isolate and extra 20% water added; 30%, egg sausage with 3% modified cassava starch and 1% whey protein isolate and extra 30% water added.

The addition of 3% modified cassava starch and whey protein isolate demonstrated a more pronounced increase in breaking force for frozen egg sausage compared to the addition of whey protein alone (Table 2 and Table 4). Analysis revealed that frozen egg sausage containing whey protein isolate and 3% modified cassava starch exhibited higher rigidity compared to samples stored at 4 °C for 24 h (Table 4). Upon thawing, the protein gel matrix released a minimal amount of water. During thermal processing, the enhanced denaturation of whey protein, starch gelatinization, and their interaction with egg protein led to increased water-holding capacity through reduced protein molecular hydrophobicity. According to Table 4, the breaking point measurements in penetration tests for frozen egg sausage were lower compared to samples stored at 4 °C for 24 h. The data demonstrated that modified cassava starch, when combined with the egg protein matrix, yielded superior frozen egg protein gel structure compared to whey protein isolate alone (Table 2). While these findings indicate increased chewiness in egg sausage following frozen storage, syneresis results confirmed that the combination of 2% whey protein isolate and 3% modified cassava starch enhanced the textural properties of egg sausage post-freezing (Figure 2).

Research by Li and Yeh [29] established that meat protein undergoes denaturation at temperatures below starch gelatinization, resulting in a continuous network formation within starch/meat composite systems. Further investigations by Huang et al. [30] revealed enhanced gel strength, hardness, and chewiness characteristics in surimi gel following refrigerated storage with tapioca starch incorporation. Studies on egg protein–dextran conjugates have demonstrated their capacity to enhance protein structure stability and significantly improve emulsifying properties in protein–polysaccharide conjugates [31,32]. The observed effects of starch on frozen egg sausage’s textural properties aligned with findings from linear programming and response surface methodology studies optimizing surimi gel texture [33]. Research conducted by Kim and Lee [34] indicated that starch incorporation led to improved firmness and water-holding capacity in surimi gel. Consequently, the addition of modified cassava starch demonstrated significant effectiveness in reducing syneresis in frozen egg sausage (Figure 2).

As demonstrated in Table 5, the penetration test revealed the effects of varying soy protein isolate concentrations on the textural characteristics of egg sausage containing 3% modified cassava starch. Analysis indicated that incorporating soy protein isolate led to notable increases (*p* < 0.05) in breaking point, breaking force, and gel strength of egg sausages stored at 4 °C for 24 h. The investigation revealed a synergistic interaction between soy protein isolate and modified cassava starch within the egg protein gel matrix, as evidenced in the egg protein–soy protein isolate mixed systems (Table 5). Furthermore, the addition of modified cassava starch at 3.0% demonstrated a significant impact (*p* < 0.05) on both breaking force and breaking point of egg sausages stored at 4 °C for 24 h (Table 3 and Table 5). Comparative analysis showed enhanced rigidity in egg sausages containing 3% modified cassava starch versus those without the additive when stored at 4 °C for 24 h (Table 3 and Table 5). Moreover, frozen egg sausages exhibited superior rigidity compared to specimens stored at 4 °C for 24 h, attributable to the minimal water loss from the frozen egg sausage–starch matrix during the thawing and starch retrogradation processes. Research by Teramoto and Fuchigami [1] demonstrated that high-pressure freezing effectively minimizes textural and structural degradation in frozen egg custard gel. Their analysis revealed that conventional freezing at atmospheric pressure (−20 °C) produced the largest ice crystal formation. The post-thaw analysis indicated an increase in breaking force and rigidity of the frozen egg sausage (Table 5). However, penetration tests indicated that the breaking point values of frozen egg sausage were comparatively lower than samples stored under refrigeration at 4 °C for 24 h, as shown in Table 5. This phenomenon can be attributed to post-thaw moisture loss, which subsequently enhanced the gel strength of the frozen egg sausage, resulting in increased product firmness after frozen storage. The experimental findings indicate that the incorporation of modified cassava starch and soy protein isolate enhances the textural characteristics of egg sausage during both pre- and post-cold storage periods. According to Li et al. [35], thermal processing revealed no chemical interaction between cassava starch and soy protein concentrate. Their findings suggest that protein network modification occurs through the displacement of proteins by gelatinized starch.

Table 6 presents the analysis of texture properties in egg sausages containing 1% whey protein isolate and 3% modified cassava starch with varying water content. Statistical analysis revealed significant (*p* < 0.05) reductions across all measured parameters during penetration testing, conducted at both refrigerated (4 °C, 24 h) and frozen (−18 °C, 15 days) conditions. The data demonstrate that egg sausages containing 1% whey protein isolate and 3% modified cassava starch exhibit enhanced rigidity under frozen conditions compared to refrigerated storage at 4 °C for 24 h. This observation correlates with the decreased breaking point observed in frozen samples as water content increases. Upon thawing, a minimal quantity of water was released from the protein gel matrix. The penetration test data indicate lower breaking points in frozen samples compared to those refrigerated at 4 °C for 24 h. While frozen storage resulted in enhanced chewiness, the penetration tests demonstrated that increased water content contributed to improved tenderness and softness in both pre- and post-storage conditions (Table 6). The findings demonstrate that incorporating modified cassava starch and whey protein enhances the water retention capabilities of frozen egg sausage. As illustrated in Figure 2, the incorporation of 3% modified cassava starch and 2% whey protein isolate significantly reduces syneresis. The egg protein gelation process maintains equilibrium through a tertiary, three-dimensional network structure. This network structure effectively immobilizes substantial quantities of water when combined with whey protein isolate, modified cassava starch, and sugar, thereby enhancing the cross-linking between protein strands. The formation of covalent bonds between proteins facilitates both inter- and intra-molecular cross-linking within the egg sausage protein gels. Additionally, hydrogen bonds, hydrophobic interactions, and disulfide bonds between polymers serve crucial functions in maintaining the stability and cross-linking of the composite matrix. Despite the introduction of additional water, the combination of 3% modified cassava starch and 1% whey protein isolate demonstrated superior water retention in thawed egg sausage compared to the application of whey protein isolate independently (Figure 1B). The reinforcing effects observed in composite protein–starch gels can be attributed to the expansion of modified cassava starch granules within the egg and whey protein isolate gel matrix. This process facilitates matrix compaction, resulting in reduced syneresis and enhanced firmness [34]. Research by Tolstoguzov [36] indicates that protein–polysaccharide mixtures typically exhibit thermodynamic incompatibility at neutral pH, leading to phase separation. Subsequently, Clark [37] established that the gelation of these incompatible polymer mixtures produces structures classified as filled or composite gels [37]. In these binary mixtures, protein and starch components demonstrate the capacity to establish continuous gel networks, with network formation and strength being influenced by phase stability, concentration, and temperature conditions.

### 2.2. Sensory Evaluation of Egg Sausages at Various Protein Isolate and Modified Cassava Starch Concentrations

Table 7 presents the sensory evaluation findings for frozen egg sausages formulated with modified cassava starch, whey protein isolate, and soy protein isolate. The assessment revealed that egg sausages incorporating 0.5% whey protein isolate demonstrated superior texture characteristics and overall acceptability compared to those containing soy protein isolate. The sensory evaluation data indicate that soy protein isolate may not be the optimal ingredient choice for egg sausage formulation. This observation can be attributed to the molecular properties of whey protein isolate, which exhibits smaller particle size and enhanced integration capability within the egg protein gel matrix compared to soy protein isolate [22].

Analysis revealed comparable springiness and overall acceptability between frozen egg sausages containing 0.5% and 1.0% whey protein isolate. Regarding the formulation containing 3% modified cassava starch, panelists found most sensory attributes satisfactory, with the exception of springiness. This reduced springiness may be attributed to the product’s elevated texture profile. Furthermore, empirical evidence suggests that the incorporation of 3% modified cassava starch and 1% whey protein isolate effectively enhances both textural properties and syneresis control in frozen products. Research indicates that whey protein isolate demonstrates superior gel elasticity at lower concentrations compared to soy protein isolate, primarily due to molecular weight variations [9]. The molecular structure of whey protein isolate exhibits a compact globular configuration with relatively small molecular weights (MW 14,000 for α-lactabumin and MW 18,000 for β-lactoglobulin), whereas soy protein isolate presents larger molecular structures (MW 140,000–190,000 for 7S globulins and MW 300,000–400,000 for 11S globulins). Consequently, these distinctions in molecular dimensions and water affinity properties appear to contribute to the enhanced sensory evaluation results observed in egg sausages containing whey protein isolate.

## 3. Conclusions

Our research demonstrates that egg sausage exhibits notable deterioration in textural properties following 15 days of frozen storage. The results indicate that no synergistic interactions were observed in egg protein–protein isolate systems at whey protein isolate and soy protein isolate concentrations ranging from 0.5% to 2%. The research establishes that incorporating 0.5–1.0% whey protein isolate and 3% modified cassava starch substantially enhances both syneresis control and textural attributes in frozen egg sausages. This improvement is attributed to the stabilizing effect of the gelled matrix formed by whey protein isolate and modified cassava starch, which proves more effective than the combination of soy protein isolate and whey protein isolate. Sensory evaluation data confirm that frozen egg sausages containing 0.5–1.0% whey protein isolate received superior ratings compared to formulations with other protein and starch combinations. Furthermore, the addition of 3.0% modified cassava starch effectively mitigates texture degradation post-thawing and improves syneresis control. It is noteworthy that formulations containing soy protein isolate at 0.5% concentration demonstrated lower acceptability in frozen product sensory evaluations. Subsequent research directions may investigate the efficacy of various polysaccharides, including gellan gum, carrageenan, and agar, in enhancing textural properties and reducing syneresis in frozen and thawed egg sausages. Moreover, the implementation of sophisticated analytical methodologies could provide deeper insights into water-binding mechanisms and syneresis control, utilizing techniques such as FT-IR spectroscopy, UV-VIS spectroscopy, nuclear magnetic resonance spectroscopy, optical imaging, differential scanning calorimetry, and scanning electron microscopy.

## 4. Materials and Methods

### 4.1. Raw Materials

Soy protein isolate (Shandong Wucheng Dawang group Co., Ltd., Qingdao, China) and Modified cassava starch (CLEARAM^®^ T-1, ROQUETTE, Merville, France) were provided by Gemfont Corporation (Taipei, Taiwan). Whey protein isolate (HG-80) was purchased from Yi Yuan Food Corporation (Keelung, Taiwan). Whole eggs were purchased from Yun Chen Egg Production Corporation (LinKou, Taipei, Taiwan). Sucrose was purchased from Taiwan Sugar Corporation (Siaogang, Kaohsiung, Taiwan). Salt was obtained from Taiyen Corporation (Tainan, Taiwan). Nalo fibrous artificial casings were purchased from Godja Tech Corporation (Taipei, Taiwan).

### 4.2. Preparation of Egg Sausages

The whole egg mixture consisted of 1000 g egg, 15 g sucrose, and 20 g salt. Modified cassava starch was incorporated at 3% of the whole egg weight (10 g to 30 g per 1000 g of egg). Mixtures of whey protein isolate (5 g to 10 g) and soy protein isolate (5 g to 10 g) at various concentration levels were blended at 1200 rpm for 5 min in a Robot Coupe BLIXE 3 (Jackson, MS, USA) and put into a refrigerator for 10 min. The bubble on the top was scraped off. The blended mixtures were poured into 25 mm diameter cellulose casings (Godja Tech Co., Ltd., Taipei, Taiwan). Each egg sausage was 100 g and 10 cm in length. Then, the egg sausages were sealed and simmered at 85 °C for 20 min. Cooked egg sausages were cooled to room temperature, and half of cooked egg sausages were stored at 4 °C for 24 h to evaluate the textural properties. Half of cooked egg sausages were frozen at −25 °C in a cold chamber with forced-air convection and stored at −18 °C for 15 days. Samples were thawed to room temperature to evaluate textural properties, syneresis, and sensory evaluation. The textural properties and % syneresis of egg sausages were recorded before and after refrigerator storage for 1 day and left the egg sausages back to room temperature.

### 4.3. Measurement of Textural Properties

Penetration test of egg sausages was carried out using a texture analyzer (Model TA-XT2, Stable Micro Ltd., Aaslemere, UK) at room temperature. Ten slices of egg sausage samples were cut straight with a uniform geometry (1 cm high × 2.5 cm diameter). Each sample underwent breaking force and breaking point measurement using a spherical plunger (5 mm diameter), with a depression speed of 100 mm/min to a depth of 10 mm. For calibration, a 5 kg load cell was employed, with a trigger force of 5 g. Gel strength and rigidity were calculated using the following equation [38,39].

Gel strength (N × mm) = breaking force (N) · breaking point (mm)(1)

(2) $$\mathrm{Rigidity}\;(\frac{N}{mm})\;=\;\frac{breaking\;force\;(N)}{breaking\;point\;(mm)}$$

Measurements were carried out at room temperature (25–27 °C) on duplicates. Each datum was the mean of 10 determinations.

### 4.4. Evaluation of Syneresis of Frozen Egg Sausages

The method suggested by Charoenrein et al. [40] was used with modifications to determine the syneresis of frozen egg sausages. All egg sausages were stored at −18 °C for 15 days within plastic boxes. Before storage the samples were weighed (w1) by analytical balance (Model ATX224, Shimadzu Corporation, Tokyo, Japan). The separated water was wiped off with tissue paper and weighed (w2) again after 15 days of storage in freezer and then thawed to room temperature. Syneresis of egg sausage was calculated as (w1 − w2)/w1 and expressed as percent basis.

### 4.5. Sensory Evaluation

Twenty-five volunteers (twelve females and thirteen males, aged 16–65) were recruited from the Department of Food Science, National Taiwan Ocean University, for sensory testing of egg sausages. The participants, comprising students, staff, and faculty members, had prior experience in sensory analysis experiments [41] and were familiar with egg sausages. A preliminary training session was conducted to familiarize panelists with the important attributes of egg sausages and to establish a standardized hedonic scale for evaluation. The panelists were trained to assess four key attributes of egg sausages: springiness, firmness, chewiness, and overall acceptability. Springiness was defined as the degree of springiness in egg sausage by pressing between fingers and measuring the recovery time. Firmness was characterized as the force required to eliminate the resistance and bite completely through egg sausage placed between the molars on the first bite. Upon mastering these texture attributes, the panelists proceeded to evaluate the egg sausage samples. Samples were cooked at 85 °C for 20 min, sufficient to reach 75 °C in the interior of the sample. This temperature was measured with a thermometer. Two slices of each sample (20 mm high approximately) were served and identified by three-digit random code. A hedonic 7-point scale in which the panelists evaluated different attributes: springiness (1 = dislike very much, 7 = like very much), texture (1 = dislike very much, neither like nor dislike the firmness, 7 = like very much), and overall acceptability (1 = dislike very much, 4 = neither like nor dislike, 7 = like very much). Panelists evaluated the egg sausages in a control testing area. Water and unsalted crackers were provided to clean the palate between samples to minimize any residual effect. Each datum was the mean of twenty-five determinations.

### 4.6. Statistical Analysis

All results were tested using analysis of variance techniques (ANOVA). The difference between means at a 5% significance level (*p* < 0.05) was determined using Ducan’s multiple range test. All statistical analyses were performed using the Statistics Package for Social Science 12.0 software for Windows (SPSS, 2004). All results were presented as the mean ± standard deviation.

## Figures and Tables

**Table 1:** Effect of modified cassava starch amounts on textural characteristics ^1^ of egg sausage during cold storage (mean ± SD, *n* = 10).

Modified Cassava Starch (%)	Breaking Point	Breaking Force	Gel Strength	Rigidity
	(mm)	(N)	(N × mm)	(N/mm)
24 h at 4 °C				
0 (Control)	9.60 ± 0.16 ^a^	21.94 ± 0.40 ^c^	210.8 ± 6.9 ^a^	2.28 ± 0.03 ^d^
1.0	9.06 ± 0.23 ^b^	20.81 ± 0.45 ^c^	188.1 ± 7.9 ^b^	2.29 ± 0.01 ^d^
2.0	8.25 ± 0.10 ^c^	23.52 ± 0.18 ^b^	194.1 ± 2.6 ^b^	2.85 ± 0.05 ^c^
3.0	8.16 ± 0.23 ^c^	25.60 ± 1.22 ^a^	209.2 ± 15.7 ^a^	3.13 ± 0.06 ^b^
15 Days at −18 °C				
0	6.43 ± 0.41 ^e^	24.54 ± 1.83 ^b^	157.8 ± 13.1 ^c^	3.83 ± 0.43 ^a^
1.0	6.84 ± 0.24 ^d^	23.87 ± 0.86 ^b^	163.3 ± 7.5 ^c^	3.49 ± 0.19 ^a^
2.0	7.07 ± 0.28 ^d^	26.57 ± 1.65 ^a^	184.9 ± 2.4 ^b^	3.75 ± 0.13 ^a^
3.0	7.18 ± 0.18 ^d^	26.71 ± 0.77 ^a^	192.0 ± 8.6 ^b^	3.71 ± 0.12 ^a^

^1^ Gel strength = (Breaking force × Breaking point); Rigidity = (Breaking force/Breaking point); Mean with a different letter in the same sub-column are significantly different (*p <* 0.05).

**Table 2:** Effect of whey protein isolate concentrations on textural characteristics ^1^ of egg sausage during cold storage (mean ± SD, *n* = 10).

Whey Protein Isolate (%)	Breaking Point	Breaking Force	Gel Strength	Rigidity
	(mm)	(N)	(N × mm)	(N/mm)
24 h at 4 °C				
0 (Control)	9.60 ± 0.16 ^b^	21.94 ± 0.40 ^a^	210.8 ± 6.9 ^b^	2.28 ± 0.03 ^a^
0.5	8.63 ± 0.33 ^a^	22.10 ± 1.04 ^a^	191.1 ± 15.3 ^a^	2.55 ± 0.08 ^b^
1	9.37 ± 0.17 ^b^	23.69 ± 1.25 ^b^	222.1 ± 14.7 ^b^	2.52 ± 0.11 ^b^
15 Days at −18 °C				
0	6.43 ± 0.41 ^d^	24.54 ± 1.83 ^b^	157.8 ± 13.1 ^d^	3.83 ± 0.43 ^d^
0.5	5.97 ± 0.18 ^c^	17.43 ± 0.35 ^c^	104.2 ± 3.8 ^c^	2.91 ± 0.10 ^c^
1	6.02 ± 0.18 ^d^	18.35 ± 0.85 ^c^	110.4 ± 4.3 ^c^	3.05 ± 0.20 ^c^

^1^ Gel strength = (Breaking force × Breaking point); Rigidity = (Breaking force/Breaking point); Mean with a different letter in the same sub-column are significantly different (*p* < 0.05).

**Table 3:** Effect of soy protein isolate concentrations on textural characteristics ^1^ of egg sausage during cold storage (mean ± SD, *n* = 10).

Soy Protein Isolate (%)	Breaking Point	Breaking Force	Gel Strength	Rigidity
	(mm)	(N)	(N × mm)	(N/mm)
24 h at 4 °C				
0 (Control)	9.60 ± 0.16 ^ab^	21.94 ± 0.40 ^b^	210.8 ± 6.9 ^b^	2.28 ± 0.03 ^ab^
0.5	9.84 ± 0.39 ^b^	21.32 ± 0.55 ^a^	209.7 ± 5.3 ^ab^	2.16 ± 0.12 ^a^
1	9.29 ± 0.25 ^a^	21.68 ± 0.24 ^ab^	201.5 ± 5.4 ^a^	2.33 ± 0.07 ^b^
15 Days at −18 °C				
0	6.43 ± 0.41 ^c^	24.54 ± 1.83 ^b^	157.8 ± 13.1 ^c^	3.83 ± 0.43 ^c^
0.5	6.60 ± 0.29 ^c^	16.39 ± 0.66 ^c^	108.2 ± 5.0 ^d^	2.48 ± 0.17 ^b^
1	6.21 ± 0.22 ^c^	15.20 ± 1.04 ^c^	94.3 ± 4.2 ^e^	2.45 ± 0.25 ^b^

^1^ Gel strength = (Breaking force × Breaking point); Rigidity = (Breaking force/Breaking point); Mean with a different letter in the same sub-column are significantly different (*p* < 0.05).

**Table 4:** Effect of whey protein isolate concentrations on textural characteristics ^1^ of egg sausage containing 3% modified cassava starch during cold storage (mean ± SD, *n* = 10).

Whey Protein Isolate (%)	Breaking Point	Breaking Force	Gel Strength	Rigidity
	(mm)	(N)	(N × mm)	(N/mm)
24 h at 4 °C				
0	8.16 ± 0.23 ^a^	25.6 ± 1.2 ^b^	209.2 ± 15.7 ^a^	3.13 ± 0.06 ^c^
1	9.53 ± 0.10 ^b^	24.5 ± 0.3 ^a^	233.6 ± 2.1 ^b^	2.57 ± 0.08 ^a^
2	9.86 ± 0.07 ^c^	28.7 ± 0.2 ^c^	283.0 ± 3.5 ^c^	2.91 ± 0.04 ^b^
15 Days at −18 °C				
0	7.18 ± 0.18 ^d^	26.7 ± 0.7 ^b^	192.0 ± 8.6 ^a^	3.71 ± 0.12 ^d^
1	7.89 ± 0.45 ^a^	28.6 ± 1.7 ^c^	225.8 ± 14.0 ^b^	3.62 ± 0.39 ^d^
2	8.14 ± 0.16 ^a^	31.5 ± 0.8 ^d^	257.0 ±11.4 ^d^	3.87 ± 0.04 ^d^

^1^ Gel strength = (Breaking force × Breaking point); Rigidity = (Breaking force/Breaking point); Mean with a different letter in the same sub-column are significantly different (*p* < 0.05).

**Table 5:** Effect of soy protein isolate concentrations on textural characteristics ^1^ of egg sausage containing 3% modified cassava starch during cold storage (mean ± SD, *n* = 10).

Soy Protein Isolate (%)	Breaking Point	Breaking Force	Gel Strength	Rigidity
	(mm)	(N)	(N × mm)	(N/mm)
24 h at 4 °C				
0	8.16 ± 0.23 ^a^	25.6 ± 1.2 ^a^	209.2 ± 15.7 ^a^	3.13 ± 0.06 ^b^
1	9.45 ± 0.41 ^b^	27.7 ± 0.9 ^b^	261.8 ± 4.3 ^b^	2.93 ± 0.41 ^a^
2	9.88 ± 0.13 ^c^	33.8 ± 0.6 ^c^	334.5 ± 1.7 ^c^	3.42 ± 0.13 ^c^
15 Days at −18 °C				
0	7.18 ± 0.18 ^e^	26.7 ± 0.7 ^a^	192.0 ± 8.6 ^a^	3.71 ± 0.12 ^d^
1	7.57 ± 0.45 ^d^	30.6 ± 1.7 ^d^	231.6 ± 14.0 ^d^	4.04 ± 0.39 ^e^
2	7.89 ± 0.08 ^a^	34.7 ± 0.4 ^c^	273.8 ± 5.5 ^e^	4.00 ± 0.04 ^e^

^1^ Gel strength = (Breaking force × Breaking point); Rigidity = (Breaking force/Breaking point); Mean with a different letter in the same sub-column are significantly different (*p* < 0.05).

**Table 6:** Effect of water addition on textural characteristics ^1^ of egg sausage containing 3% modified cassava starch and 1% whey protein isolate during cold storage (mean ± SD, *n* = 10).

Water Addition (%)	Breaking Point	Breaking Force	Gel Strength	Rigidity
	(mm)	(N)	(N × mm)	(N/mm)
24 h at 4 °C				
0	9.53 ± 0.10 ^c^	24.5 ± 0.3 ^d^	233.6 ± 2.1 ^d^	2.57 ± 0.08 ^b^
10	7.64 ± 0.19 ^b^	20.1 ± 0.4 ^c^	153.7 ± 5.6 ^c^	2.63 ± 0.07 ^b^
20	7.46 ± 0.08 ^b^	15.8 ± 0.3 ^b^	117.9 ± 1.9 ^b^	2.11 ± 0.06 ^a^
30	6.58 ± 0.08 ^a^	13.4 ± 0.1 ^a^	88.6 ± 1.3 ^a^	2.04 ± 0.03 ^a^
15 Days at −18 °C				
0	7.89 ± 0.45 ^b^	28.6 ± 1.7 ^f^	225.8 ± 14.0 ^d^	3.62 ± 0.39 ^d^
10	5.89 ± 0.53 ^d^	20.8 ± 1.5 ^c^	123.6 ± 19.8 ^b^	3.54 ± 0.09 ^d^
20	5.67 ± 0.08 ^d^	17.0 ± 0.1 ^e^	96.8 ± 1.5 ^f^	3.00 ± 0.05 ^c^
30	4.51 ± 0.22 ^e^	13.7 ± 0.9 ^a^	61.8 ± 6.8 ^e^	3.03 ± 0.17 ^c^

^1^ Gel strength = (Breaking force × Breaking point); Rigidity = (Breaking force/Breaking point); Mean with a different letter in the same sub-column are significantly different (*p* < 0.05).

**Table 7:** Sensory evaluation of frozen egg sausages containing different polysaccharides (mean ± SD, *n* = 25).

Frozen Egg Sausages ^1^	Springiness	Texture	Overall Acceptability
1.0% Modified cassava starch	3.68 ± 0.74 ^ab^	3.96 ± 0.78 ^ab^	3.76 ± 0.43 ^a^
2.0% Modified cassava starch	4.48 ± 0.50 ^b^	3.76 ± 0.43 ^a^	3.84 ± 0.47 ^a^
3.0% Modified cassava starch	3.20 ± 0.40 ^ab^	5.20 ± 0.40 ^c^	4.88 ± 0.43 ^b^
0.5% Whey protein isolate	4.80 ± 0.50 ^c^	4.80 ± 0.40 ^c^	5.48 ± 0.50 ^b^
1.0% Whey protein isolate	4.68 ± 0.47 ^bc^	4.36 ± 0.56 ^b^	5.32 ± 0.69 ^b^
0.5% Soy protein isolate	4.76 ± 0.52 ^bc^	4.32 ± 0.47 ^b^	3.60 ± 0.64 ^a^
1.0% Soy protein isolate	4.44 ± 0.71 ^b^	3.88 ± 0.78 ^a^	3.80 ± 0.57 ^a^

^1^ A hedonic 7-point scale in which the panelists evaluated different attributes: springiness (1 = dislike very much, 7 = like very much), texture (1 = dislike very much, neither like nor dislike the texture, 7 = like very much), and overall acceptability (1 = dislike very much, 4 = neither like nor dislike, 7 = like very much); mean with a different letter in the same sub-column are significantly different (*p* < 0.05).

## Data Availability

The data presented in this work are available upon request from the corresponding author.
